# Neutrophil extracellular traps in central nervous system pathologies: A mini review

**DOI:** 10.3389/fmed.2023.1083242

**Published:** 2023-02-17

**Authors:** Areez Shafqat, Ahmed Noor Eddin, Ghaith Adi, Mohammed Al-Rimawi, Saleha Abdul Rab, Mylia Abu-Shaar, Kareem Adi, Khaled Alkattan, Ahmed Yaqinuddin

**Affiliations:** College of Medicine, Alfaisal University, Riyadh, Saudi Arabia

**Keywords:** neutrophil extracellular traps, blood-brain barrier, neuroinflammation, stroke, neurodegeneration, traumatic brain injury, Alzheimer’s disease, neutrophils (PMNs)

## Abstract

Neutrophils are the first cells to be recruited to sites of acute inflammation and contribute to host defense through phagocytosis, degranulation and neutrophil extracellular traps (NETs). Neutrophils are rarely found in the brain because of the highly selective blood-brain barrier (BBB). However, several diseases disrupt the BBB and cause neuroinflammation. In this regard, neutrophils and NETs have been visualized in the brain after various insults, including traumatic (traumatic brain injury and spinal cord injury), infectious (bacterial meningitis), vascular (ischemic stroke), autoimmune (systemic lupus erythematosus), neurodegenerative (multiple sclerosis and Alzheimer’s disease), and neoplastic (glioma) causes. Significantly, preventing neutrophil trafficking into the central nervous system or NET production in these diseases alleviates brain pathology and improves neurocognitive outcomes. This review summarizes the major studies on the contribution of NETs to central nervous system (CNS) disorders.

## Introduction

**FIGURE 1:**
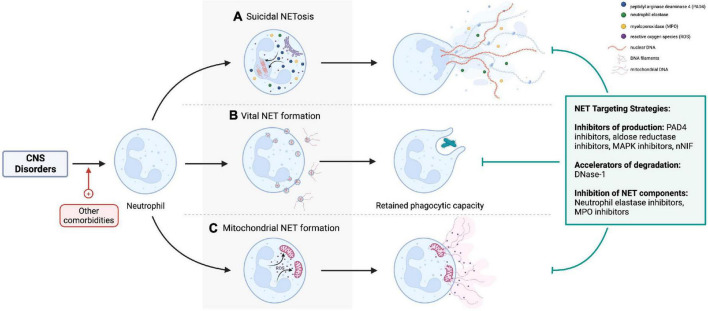
**(A)** Lytic NETosis describes neutrophil cell death with release of intracellular contents as neutrophil extracellular traps (NETs). By contrast, neutrophils retain viability and phagocytic effector functions after **(B)** vital and **(C)** mitochondrial NET formation. Nicotinamide adenine dinucleotide phosphate oxidase activation, reactive oxygen species, neutrophil elastase (NE), myeloperoxidase (MPO), and peptidyl-arginine deaminase-4 (PAD-4) are important cellular mediators of NETs production. NETs can be targeted pharmacologically by preventing their formation (e.g., PAD4 inhibitors, NE inhibitors), accelerating degradation by DNase, or inhibiting specific NET components [e.g., NE inhibitors, matrix metalloproteinase-9 (MMP-9) inhibitors]. Created with BioRender.com.

Loss of blood-brain barrier (BBB) integrity and neuroinflammation are central to the pathogenesis of central nervous system (CNS) pathologies. Neutrophils are the most abundant leukocyte population, which adhere to activated endothelium, transmigrate to tissues, and contribute to inflammatory processes. Neutrophil extracellular traps (NETs) produced by activated neutrophils are composed of a DNA scaffold on which various proteins are deposited (1). The release of NETs was traditionally related to neutrophil death (termed NETosis), but neutrophils also undergo vital and mitochondrial NET production by extruding their contents through blebbing from the cell membrane (2), remaining viable and retaining effector functions (Figure 1).

The production of different types of NETs is context-dependent, varying with the nature of the NET-inducing stimuli and disease process (3). For example, lytic NETosis is induced by infections (4), pro-inflammatory cytokines (5), damage-associated molecular patterns (DAMPs) (6, 7), activated platelets (8, 9), complement (10, 11), autoantibodies (12, 13), and immune complexes; vital NETs are mainly seen in infections (2, 14, 15); and mitochondrial NETs during infections and autoimmune diseases (16, 17). The molecular mechanisms governing NET production in each of these instances are beyond the scope of this review and are discussed elsewhere (18–20). Regulators of this heterogeneity of NETs and whether different types of NETs exert variable, context-specific effects (beneficial versus harmful) are outstanding questions in the field of NETs research (21).

Despite their apparent benefit in infectious disease (4), NETs cause significant collateral tissue damage (22). For example, cell-free DNA (16), histones (23–25), matrix metalloproteinase-9 (MMP-9) (26), and LL-37 (27), have all been implicated in various tissue-damaging effects, including direct cytotoxicity (28), thrombosis (29), and chronic inflammation (24). Several excellent reviews on NETs in defense and disease have been published (30–36). Recent studies have shown neutrophil recruitment in the CNS and NETs to contribute to various CNS pathologies. Table 1 summarizes seminal studies implicating NETs as pathogenic in brain CNS diseases. This review provides a concise summary of recent data on the role of NETs in various brain disorders.

**TABLE 1:** Evidence of neutrophil extracellular traps (NETs) in central nervous system (CNS) diseases.

Infections	CSF analysis of patients with acute pneumococcal meningitis, Lyme neuroborreliosis, and viral meningitis reveals the presence of NETs (58, 154). DNase-1 treatment significantly reduces bacterial load in the brain, liver, spleen, and blood of rat models of pneumococcal meningitis from the SP001 strain isolated from patients (58).
Trauma	Leukocyte adhesion molecules and chemoattractants in contusional and peri-contusional brain tissue facilitate neutrophil adhesion and extravasation, releasing NETs. Cl-amidine and DNase-1 reduced CNS NETs, cerebral edema, improved cerebral blood flow, and improved neurologic function post-TBI (70).
	Paroxysmal sympathetic hyperactivity (PSH) increases morbidity and mortality risk in TBI patients and correlates with NETs in the paraventricular nucleus. The protein LL-37 in NETs activates microglial cells to release IL-1β, which may mediate PSH (73).
	Neutrophil infiltration and NET production occur after spinal cord injury (SCI) in rat models, associated with neuroinflammation and spinal cord edema, and prevented by Cl-amidine and DNase-1. NETs impair blood-spinal cord barrier integrity by degrading tight junctions and upregulating TRPV4 expression on endothelial cells. NET inhibition by Cl-amidine or DNase-1 promote functional motor recovery post-SCI (155).
Stroke	In mice, CNS neutrophil recruitment to peri-infarct areas and PAD-4-dependent NET production *via* PAD-4 peaks 3–5 days after stroke. DNase-1 or Cl-amidine/PAD-4 deficiency reduce BBB permeability and increase vascular remodeling. Levels of type I IFN increased 10-fold in the ischemic cortex and were decreased by Cl-amidine, indicating NET-induced type I IFN production, increasing BBB permeability (54).
	Brain specimens from patients who died from ischemic stroke reveal dense neutrophilic infiltration and NET production in the ipsilateral brain tissue. NETs are detected in platelet-rich areas in ischemic stroke thrombi. HMGB1 expression is increased on the surface of platelets and in the plasma of stroke patients, induces NET production, and exacerbates ischemic brain injury (78).
Alzheimer’s disease	Neutrophils adhere to BMECs and extravasate into the brain of 9–13 months-old 5xFAD mice, which show Aβ accumulation in the brain. Intraparenchymal neutrophils specifically migrated toward areas of Aβ plaques (120).
	Neutrophil adhesion, extravasation, and NETs were seen in the brain areas of Aβ deposits in 5xFAD mice. Soluble Aβ induced LFA-1 integrin expression on neutrophils (causing adhesion to BMECs) and activated ROS production. Depleting neutrophils by anti-Ly6G or inhibiting LFA-1 significantly improved cognitive performance, reduced microgliosis, and lowered the load of Aβ and phosphorylated tau in 3xTg-AD mice (121).
Glioma	Immunofluorescence assays for MPO and cit-H3 demonstrate higher presence of NETs in grade IV gliomas than in grade II and III tumors. NETs activate RAGE receptors on glioma cells to enhance proliferation, migration, invasiveness, and IL-8 production *in vitro*. Tumor-derived IL-8 drives neutrophil recruitment to the tumor and NET production (138).

## Neutrophil extracellular traps disrupt the blood-brain barrier

**FIGURE 2:**
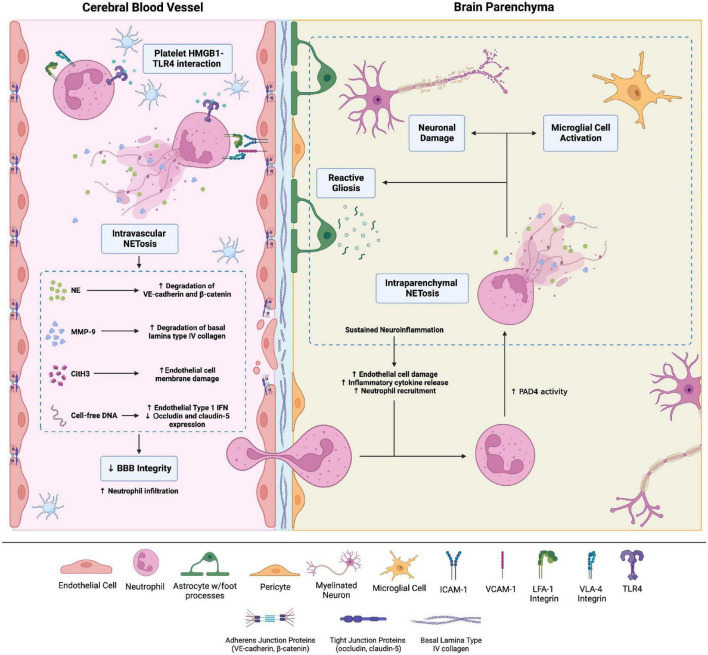
Brain injuries of various etiologies cause neutrophil adhesion to the brain microvascular endothelial cells (BMECs) *via* integrins, namely LFA-1 and VLA-4 integrins, which bind endothelial surface intercellular adhesion molecule-1 (ICAM-1) and vascular cell adhesion molecule 1, respectively. Adhesion to the BMECs activates neutrophils. Neutrophil adhesion to platelet derived high-mobility group box-1 (HMGB1) through toll-like receptor-4 (TLR4) causes neutrophil activation and intravascular neutrophil extracellular trap (NET) production. NETs comprise neutrophil elastase, citrullinated histone H3, matrix metalloproteinases (MMPs), and cell-free DNA. These NET components increase blood-brain barrier (BBB) permeability through a variety of mechanisms. For instance, MMP-9 degrades type IV collagen of the basal lamina of cerebral blood vessels, compromising blood-brain barrier (BBB) integrity. Intraparenchymal neutrophils also undergo NETosis. The ensuing neuronal damage and microglial cell activation amplify neuroinflammation and cause neuronal loss. Created with Biorender.com.

Brain microvascular endothelial cells (BMECs) are connected by various adherens and tight junctions, forming the highly selective BBB. Neutrophils thus cannot readily cross the BBB and are rarely found in a healthy brain. However, BBB permeability can increase secondary to trauma, inflammation, ischemia, and degenerative changes. Pro-inflammatory cytokines released by activated astrocytes and microglia cells upregulate adhesion molecules such as intercellular adhesion molecule-1 (ICAM-1) on BMECs, facilitating neutrophil adhesion (37). Subsequently, neutrophil-endothelial cell interactions without transmigration increase BBB permeability (Figure 2; 38).

Multiple mechanisms explain neutrophil adhesion-dependent BBB disruption. Firstly, neutrophil adhesion promotes blood flow stasis, leading to vascular obstruction; neutrophil depletion enhances CNS perfusion and decreases brain damage after stroke (39). Neutrophil adhesion to BMECs *via* the β_2_ integrins LFA-1 and MAC-1 also activates neutrophils, increasing oxidative stress and NETosis (40). Activated neutrophils release neutrophil elastase (NE)—possibly within NETs—which disrupts adherens junction proteins VE-cadherin and β-catenin, increasing BBB permeability (41). Agaphelin—an NE inhibitor—reduces BBB permeability in stroke, decreasing infarct volume, improving neurologic function, and reducing mortality in mice (42). NET-associated MMP-9 in the cerebral microvessels degrades type IV collagen of the basal lamina to disrupt BBB integrity (43). Histones also increase BBB permeability, particularly in the hippocampus, by disrupting adherens and tight junctions (44).

Extracellular DNA, microbial DNA, and damaged intracellular self-DNA are major inducers of inflammation through cGMP-AMP synthase (cGAS)/STING-dependent type I IFN and pro-inflammatory cytokine production (45–48). DNA contained within NETs also activates cGAS and enhances type I IFN production and pro-inflammatory cytokine production (16, 49). Therefore, in the CNS, NETs may promote type I IFN and pro-inflammatory cytokine responses in microglia, which are known to express cGAS and contribute to CNS pathology (50–53). NET-induced microglial activation *via* cGAS has been noted in murine models of ischemic stroke (54) and tissue plasminogen activator (tPA)-induced intracerebral hemorrhage (55). A recent preprint study demonstrated NET-induced cGAS activation in microglia in TBI, associated with neuroinflammation and neurological deficits (56). Depleting neutrophils or inhibiting peptidyl-arginine demaniase-4 (PAD-4) significantly blunt the IFN response and improve neurologic deficits (54). Together, these findings indicate that NETs mediate BBB disruption, either directly *via* histones and various proteases or indirectly by augmenting type I IFN responses.

## CNS infections

### Meningitis

Analysis of cerebrospinal fluid (CSF) from humans and rodents with pneumococcal meningitis reveals the presence of neutrophils and externalized neutrophil proteins such as myeloperoxidase (MPO), histones, NE, and proteinase 3 (PR3) (57), which may imply NET production. Combining penicillin with DNase-1 to treat pneumococcal meningitis accelerates NET degradation, enhances bacterial killing, lowers IL-1β levels, and decreases patient mortality compared to a penicillin regimen alone (58, 59).

Despite these findings, the beneficial effect of neutrophils in CNS infection must not be underestimated. Neutrophil depletion in mouse models of pneumococcal meningitis results in an elevated CSF bacterial load, higher IL-1β, lower TNF-α, and poor survival (60). Therefore, neutrophil and NET dynamics in CNS infections need to be studied further for therapeutic strategies to attenuate pathogenic functions of NETs without interfering with CNS protective mechanisms.

### Sepsis

Sepsis-induced encephalopathy has an abysmal prognosis. Lipopolysaccharide (LPS) causes neutrophil transmigration into brain parenchyma *via* CXCL1-CXCR2 interactions, whereas SB225002, a selective CXCR2 antagonist, mitigated neutrophil migration into the brain (61). However, this study did not directly explore NETs, neither were associations between SB225002 administration and improvement in neurologic parameters studied. Although NET production was not directly studied, CXCL1 stimulates ROS-dependent NET formation in COVID-19 (62), deep vein thrombosis (63), and cancer (64), and inhibiting the CXCL1-CXCR2 axis in experimental human and murine sepsis models by reparixin attenuates NET formation, multi-organ injury, and mortality (65). Sepsis-induced NET production has indeed been studied extensively in other organ systems, such as the lungs (65, 66). Mechanistically, extracellular histones exert significant pathologic effects in sepsis (23), and circulating cit-H3 significantly correlates with the severity of septic shock (67).

## CNS trauma

Traumatic brain injury (TBI) carries a high mortality risk, and complications such as cognitive impairment, memory deficits, post-traumatic stress disorder, post-traumatic encephalopathy, and neuroinflammation in survivors are frequent (68, 69). Neutrophils adhere to cerebral vessels, infiltrate hypoxic brain tissue, and produce NETs in murine models of TBIs (70, 71), associated with worse TBI outcomes, including cerebral edema, cognitive deficits, and paroxysmal sympathetic hyperactivity (72, 73). Activation of toll-like receptor-4 (TLR4) on neutrophils activates NET formation after TBI, which correlates with higher intracranial pressure (ICP), suggesting that neutrophils cause cerebral edema by NET production (70). TLR4-knockout, the NET formation inhibitor Cl-amidine, and DNase-1 reduce NET formation in the brain post-TBI and better neurologic and behavioral outcomes (70). Together, these findings indicate that NET-targeted therapies may be beneficial in alleviating hypoxia and cerebral edema after TBI.

## Stroke

Neutrophils infiltrate the CNS following cerebral ischemia (74) and correlate positively with neuronal loss, infarct size, and cognitive dysfunction (75). Circulating neutrophils from ischemic stroke patients exhibit higher NET formation than healthy control neutrophils (76). Moreover, CNS-infiltrating neutrophils in ischemic stroke patients form NETs (77, 78). Higher serum cell-free DNA levels in acute ischemic stroke patients correlate with worse clinical outcomes using the modified Rankin scale, and lower serum DNase levels are found in patients who developed stroke-associated infections (79). Platelet-derived high-mobility group box-1 (HMGB1) is a major inducer of NET production in ischemic stroke, and HMGB1-depleted attenuates NET formation after stroke and betters neurologic outcomes (78, 80). Along similar lines, treating mice with neonatal NET-inhibitory factor (nNIF) ameliorates NET production and decreased infarct size (78). NETs may also protect thrombi from degradation by tPA (81), and adding DNase-1 to tPA regimens significantly accelerates *ex vivo* lysis of stroke thrombi (82). Furthermore, NETs can contribute to tPA-induced intracerebral hemorrhage and BBB disruption (discussed above) (55). Since BBB disruption underlies the narrow therapeutic window of tPA administration (83), whether inhibiting NETs delays BBB disruption and increases the therapeutic window of tPA administration warrants further investigation.

## Autoimmunity

Neuropsychiatric manifestations of SLE (NPSLE) are commonly attributed to neuroinflammation; leukocyte infiltration precedes NPSLE-related cognitive deficits in mice (84). Neutrophils isolated from SLE patients display impaired phagocytosis, increased platelet aggregation, reactive oxygen species (ROS) production, and NET formation (85, 86). SLE skews the composition of the neutrophil compartment toward low-density granulocytes (LDGs), which readily produce NETs (87, 88). SLE patient plasma induces NET production by neutrophils isolated from healthy controls, indicating that autoantibodies and immune complexes in SLE plasma induce NET formation (16, 89). NETs released by LDGs upon stimulation by anti-ribonucleoprotein antibodies and immune complexes cause endothelial dysfunction—inducing apoptosis and impairing vasorelaxation—through components like MMP-2 (90). LDG NETs also contain various immunostimulatory proteins and autoantigens, like LL-37, IL-33, IL-17, and dsDNA, which can worsen inflammation and introduce neoepitopes, fostering an amplification of autoimmunity (91–93).

The degradation of NETs may be impaired in SLE, particularly during disease flares. Auto-antibodies against NET components—e.g., anti-dsDNA and anti-histone antibodies—are thought to protect NETs against degradation by circulating DNase (94). Furthermore, NET-activated C1q can directly inhibit DNase to prevent NET degradation (95). Genetic variations in DNase activity can also increase the risk of SLE. For example, individuals with DNASE1L3 gene mutations develop childhood SLE (96). These observations suggest that DNase-1 as a therapeutic strategy may not improve the clinical severity of SLE, as one study demonstrated in lupus nephritis patients (97).

Serum NET levels have not yet been studied as prognostic markers in NPSLE, nor has the effect of inhibiting neutrophil CNS trafficking or lowering serum and CSF NET markers on cognitive function been tested experimentally. The fundamental importance of the type I IFN response in SLE is well-established. Type I IFNs have indeed been linked to NPSLE (93, 98), but mechanistic links between type I IFNs and NPSLE manifestations are lacking. Neutrophils are perhaps major downstream mediators of the type I IFN response in SLE. They exhibit the highest expression of interferon-stimulated genes (ISGs) out of the myeloid cells (99). Type I IFNs induce mitochondrial NET formation by LDGs, and mitochondrial DNA within LDG NETs can activate cGAS-STING signaling in plasmacytoid dendritic cells to drive type I IFN production (16, 100). Therefore, whether NETs—cGAS/STING axis increases BBB permeability and neuroinflammation in NPSLE is an important question for future research.

## Neurodegenerative diseases

### Multiple sclerosis

Multiple sclerosis (MS) is a demyelinating disease of the CNS resulting in neuronal impairment. Elevated neutrophil-to-lymphocyte ratios, NETs, CXCL1, and CXCL5 are detected in blood samples of relapsing-remitting MS (RRMS) patients, but not other forms of MS (101–103). Neutrophils isolated from MS patients exhibit a primed phenotype characterized by reduced apoptosis and higher degranulation, ROS production, and NET formation (104). Males with MS show higher NETs levels than females (102), hinting at gender-specific differences in MS pathogenesis. The role of neutrophils and the contribution of various neutrophil components to MS pathogenesis have recently been reviewed (105). However, the mechanisms behind NET production in RRMS are not yet elucidated. Furthermore, NET components are yet to be physically associated with active MS lesions. Therefore, studies on NETs in MS are still in their infancy, and we caution against drawing any causal relationships.

The adaptive immune response plays a crucial role in the development of MS, particularly IL-17-secreting Th17 cells (106–108). Activation of the transcription factor retinoic acid-related orphan receptor γt (RORγt) is key to the differentiation of Th cells into Th17 cells and subsequent IL-17 production. Activators of RORγt have thus been the subject of intense research, as they constitute therapeutic targets to mitigate the Th17 response and ameliorate MS severity. Wilson et al. (109) recently demonstrated that NETs-derived histones promote Th17 cell differentiation and IL-17 production by engaging TLR2 on the surface of undifferentiated Th cells and activating RORγt. The histone inhibitor mCBS abrogated histone-induced Th17 differentiation (109). These mechanistic links need to be investigated in experimental models of MS; whether histone inhibition mitigates the Th17 response and betters MS outcomes is important to determine. Other components of the pathological CNS environment in MS, such as members of the chondroitin sulfate proteoglycan (CSPG) family, also promote Th17 polarization, which are toxic to oligodendrocyte precursor cells and impair remyelination (110). These data open new avenues to explore the potential interactions of innate immune cells such as neutrophils with extracellular matrix components and their collective impact in shaping the T-cell differentiation phenotype.

### Alzheimer’s disease

Alzheimer’s disease is a debilitating form of dementia and a significant cause of patient and caregiver morbidity and health expenditures. Although amyloid-β (Aβ) deposition is a pathologic hallmark of AD, amyloid-targeting therapies have been largely ineffective in treating or delaying AD progression in clinical trials (111). Therefore, new biomarkers and treatment strategies against AD are needed.

Neuroinflammation has been demonstrated to be a hallmark feature of AD and contributes to disease progression (112–115). Recent advances in understanding neutrophil biology have reignited interest in their role in AD. Initial studies demonstrated that circulating neutrophils in AD upregulate CD11b and ROS production, both markers of neutrophil activation (116). Furthermore, neutrophils are seen in the brains of AD patients and correlate with the burden of neurofibrillary tangles and Aβ (117). Neutrophils isolated from AD patients exhibit a gene expression signature indicative of mitochondrial dysfunction, energy hypometabolism, leukocyte adhesion, and cytokine signaling, suggesting that neutrophils contribute to neuroinflammation in AD (117, 118). Lastly, the role of neutrophil granule proteins—which can be found in NETs—in AD has recently been reviewed by Stock et al. (119).

Studies using 3xFAD and 3xTg AD mouse models have provided further insights into neutrophils and NETs in AD (119). Neutrophils adhere to BMECs in AD, transmigrate into the brain parenchyma and co-localize with Aβ plaques in 5xFAD and 3xTg-AD mice (120, 121). Neutrophil accumulation in AD mice brains precedes cognitive dysfunction, indicating that neutrophil recruitment contributes to AD symptomatology. Mechanistically, Aβ induces LFA-1 expression on neutrophils, responsible for their adhesion to BMECs and subsequent extravasation (121). Subsequently, Aβ amyloid fibrils from various sources induce nicotinamide adenine dinucleotide phosphate-oxidase-dependent lytic NETosis (122, 123). NE within NETs can then cleave amyloid fibrils and form cytotoxic oligomers (122). In short, these findings indicate that Aβ mediates neutrophil recruitment and transmigration through upregulation of LFA-1 on neutrophils and subsequently stimulates NETosis, the components of which can fragment Aβ to oligomers that exert neurotoxic or neuroinflammatory effects. LFA-1 blockade reduces CNS neutrophil recruitment, microgliosis, and Aβ load in the brain, and improves long-term memory (121). Intriguingly, a case report administered DNase-1 to an AD patient and noticed considerable cognitive improvement (124).

Amyloid-β may also induce intravascular and intraparenchymal NETosis through indirect mechanisms by fostering a neuroinflammatory microenvironment (125). For example, Aβ activates NLRP3 inflammasome signaling in microglial cells and the release of IL-1β, which has been demonstrated to induce NETosis in cancer (126) and gout (127, 128). However, while aberrant innate immune responses in AD were well documented, their direct relation to AD neurodegeneration was only recently shown (129). In AD, Aβ co-localizes with microglia and is endocytosed, subsequently damaging mitochondrial DNA and causing double-stranded DNA breaks (130, 131). Damaged DNA activates cGAS and promotes downstream type I IFN and pro-inflammatory cytokine responses (129). These proinflammatory, M1 microglia in turn foster neurotoxic astrocyte phenotypes, which contribute to neurodegeneration (129). Furthermore, BMECs in AD upregulate type I IFN receptors and interferon-stimulated genes, associated with downregulation of VE-cadherin and tight junction proteins occludin and claudin-5, increasing BBB leakiness (132), indicating that microglia-derived IFNs disrupt the BBB. Like microglia, neutrophils also co-localize with Aβ plaques, associated with NETosis (121, 125). Furthermore, DNA within NETs is recognized by cGAS in microglia (discussed above). On this basis, we propose that a positive feedback loop may exist, where Aβ and microglia-initiated neuroinflammation mediate neurotoxicity through astrocytes but also recruit and activate neutrophils to release NETs, that, in turn, worsen microglial activation. Testing such associations could implicate NET inhibition or degradation as a viable strategy to attenuate AD pathology and symptoms.

## Glioma

Glioma tumor cells produce granulocyte colony-stimulating factor that drives granulopoiesis in the bone marrow (133, 134). Consequently, neutrophilia and an elevated neutrophil-to-lymphocyte ratio are commonly seen in glioma patients (135), are more pronounced in high-grade (IV) tumors, and confer a poor prognosis (136). In agreement with these findings, depleting neutrophils by using an anti-Ly6G monoclonal antibody prolongs survival in mice with gliomas (137). Glioma cells also produce IL-8 that recruits neutrophils, termed tumor-associated neutrophils (TANs) (138). IL-8 induces NETosis in TANs through PI3K-signaling and ROS production. Indeed, TANs and NETs have been visualized within glioma lesions (138). NET components such as HMGB1 bind RAGE receptors on the surface of glioma tumor cells and stimulate proliferation, invasion, and IL-8 production *in vitro* (138). These findings suggest that a self-reinforcing NETs/IL-8 axis may collectively amplify tumor inflammation in glioma. In glioblastoma multiforme (GBM), TANs stimulate GBM tumor cell proliferation and epithelial-to-mesenchymal transition through S100A4 (139). NETs also contribute to a hypercoagulable state in high-grade glioma by inducing endothelial cell dysfunction, alleviated by DNase1 + protein C treatment *in vitro* (140). Given these findings, exploring specific pro-tumorigenic NET components in glioma may reveal novel disease markers and therapeutic targets in glioma. However, it is important to mention that neutrophils also exert numerous anti-tumor functions by killing tumor cells and T enhancing anti-tumor -cell responses (141). Therefore, the dilemma of inhibiting NETs and inadvertently attenuating beneficial—in this case, tumor suppressive—neutrophil functions must be carefully considered. Only rigorous mechanistic analyses can dissect beneficial versus detrimental effects of NETs in the context of cancer. We refer readers to in-depth reviews on neutrophils, NETs and cancer for more information (142–147).

Considerable data exist on TANs and NETs as mediators of chemotherapy, immunotherapy, and radiotherapy resistance (148–150). In glioma, NET-derived S100A4 mediates resistance to anti-VEGF therapy, whereas inhibition of S100A4 enhances response to treatment (139). Silencing S100A4 gene expression in endothelial cells by S100A4 small-interfering RNA induces an anti-angiogenic gene signature, and administering S100A4 siRNA into human prostate cancer xenografts significantly decreased tumor vascularity and inhibited tumor growth (151). These studies have set the stage for S100A4-targeted anti-cancer therapies. Since S100A4 may be derived from NETs, future research must ascertain whether inhibiting NET production normalizes the tumor vasculature and constitutes a potential anti-angiogenic cancer treatment strategy.

## Conclusion

Single-cell technologies have revealed that the neutrophil population comprises functionally distinct subsets. Understanding how the neutrophil population is skewed in CNS diseases is essential to identifying context-dependent neutrophil transcriptomic alterations and novel disease-specific therapeutic strategies. Therefore, the composition and function of NETs may also vary in different CNS diseases; lytic NETs, vital NETs, and mitochondrial NETs are released in response to distinct stimuli and have varying compositions. However, studies investigating NETs in CNS disease thus far do not account for NET heterogeneity; the specific type of NETs seen are rarely reported. Therefore, if NETs are to be considered a future therapeutic target in CNS disease, they need to be studied in greater resolution. Future studies into the topic may reveal novel disease markers and cause-specific therapeutic targets to improve the care of patients suffering from CNS diseases. Interestingly, while the activation of canonical and non-canonical inflammasome pathways was previously linked to NETosis (152), a recent study showed that inflammasome-related NET formation does not require cell death (153). Therefore, the field of NETs research is wide open; new research will likely reveal new NET-inducing stimuli, important signaling pathways worth targeting to mitigate NET formation, new NET components, and yet unknown beneficial and harmful effects.

## Author contributions

AS and AY: conceptualization. AS, ANE, GA, MA-R, SAR, and KAd: writing—original draft preparation. AS, KAd, and AY: writing—review and editing. KAd and AY: supervision. All authors have read and agreed to the published version of the manuscript.
